# Supplementary material for: Dereplication of microbial metabolites through database search of mass spectra
Source: Nat Commun. 2018 Oct 2;9:4035. doi: 10.1038/s41467-018-06082-8 (PMC6168521; doi:10.1038/s41467-018-06082-8)
Supplement: Supplementary file 1 — Supplementary Information [file 41467_2018_6082_MOESM1_ESM.pdf]

SUPPLEMENTARY INFORMATION

Dereplication of Microbial Metabolites Through Database Search of Mass Spectra

Mohimani et al.

SUPPLEMENTARY FIGURES

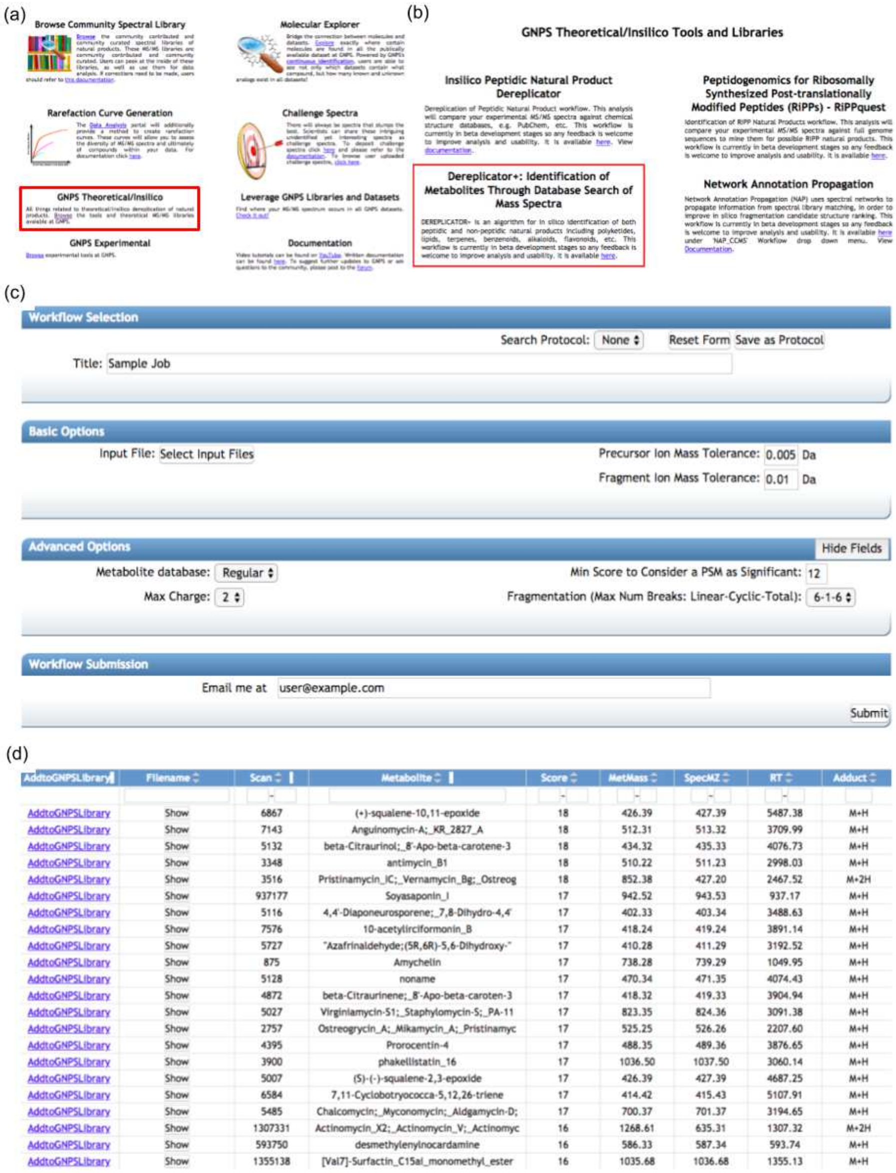

Supplementary Figure 1: Running DEREPLICATOR+ on the GNPS web server. (a) Image of the GNPS web page at www.gnps.edu. DEREPLICATOR+ users click on "Browse" in the section "GNPS Theoretical/Insilico" (b) At "DEREPLICATOR+, Identification of Metabolites Through Database Search of Mass Spectra" section, users click on "here". (c) To select input files, users click on "Select Input Files". Users can either import one of the existing GNPS public datasets from the "Share Files" section, or use their own data from "Upload Files" section. Users select a title/email for the job and specify the DEREPLICATOR+ accuracy mode (precursor and fragment ion mass tolerances). Users receive a notification email after the job is completed. (d) When the job is completed, users click on "View Significant Matches" to see all the identifications. Further guidelines and information on using DEREPLICATOR+ are available at: "https://bix-lab.ucsd.edu/display/Public/Insilico+Natural+Products+Dereplicator+Documentation" . The search results for $\mathrm{Spectra}_{\mathrm{ActiSeq}}$, $\mathrm{Spectra}_{\mathrm{Cyan}}$, and $\mathrm{Spectra}_{\mathrm{Lichen}}$ are available at "https://gnps.ucsd.edu/ProteoSAFe/status.jsp?task=5ea18d0c3b1d4018b7e0e79445ca8c18", "https://gnps.ucsd.edu/ProteoSAFe/result.jsp?task=95ebc1c169654d838c41df41cc24f88a", and "https://gnps.ucsd.edu/ProteoSAFe/status.jsp?task=00c03bdbdae644e4935a8e094f249c2a" respectively.

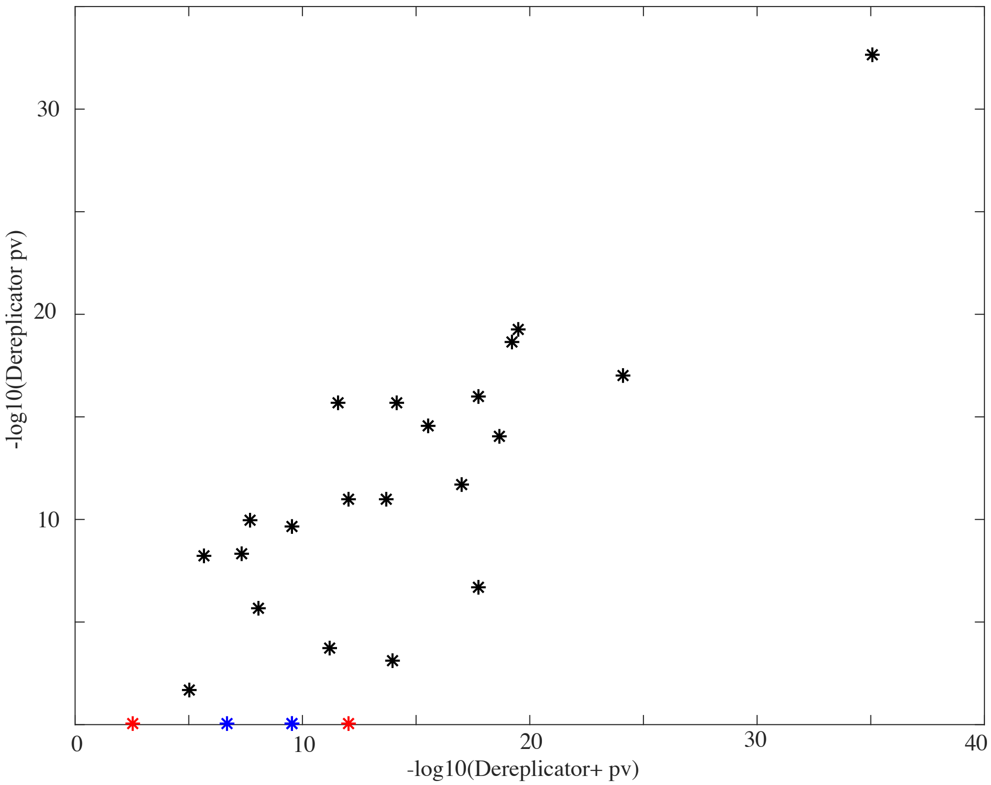

Supplementary Figure 2: DEREPLICATOR p-values versus DEREPLICATOR+ p-values. Peptides are shown in black, lipids in blue and polyketides in red.

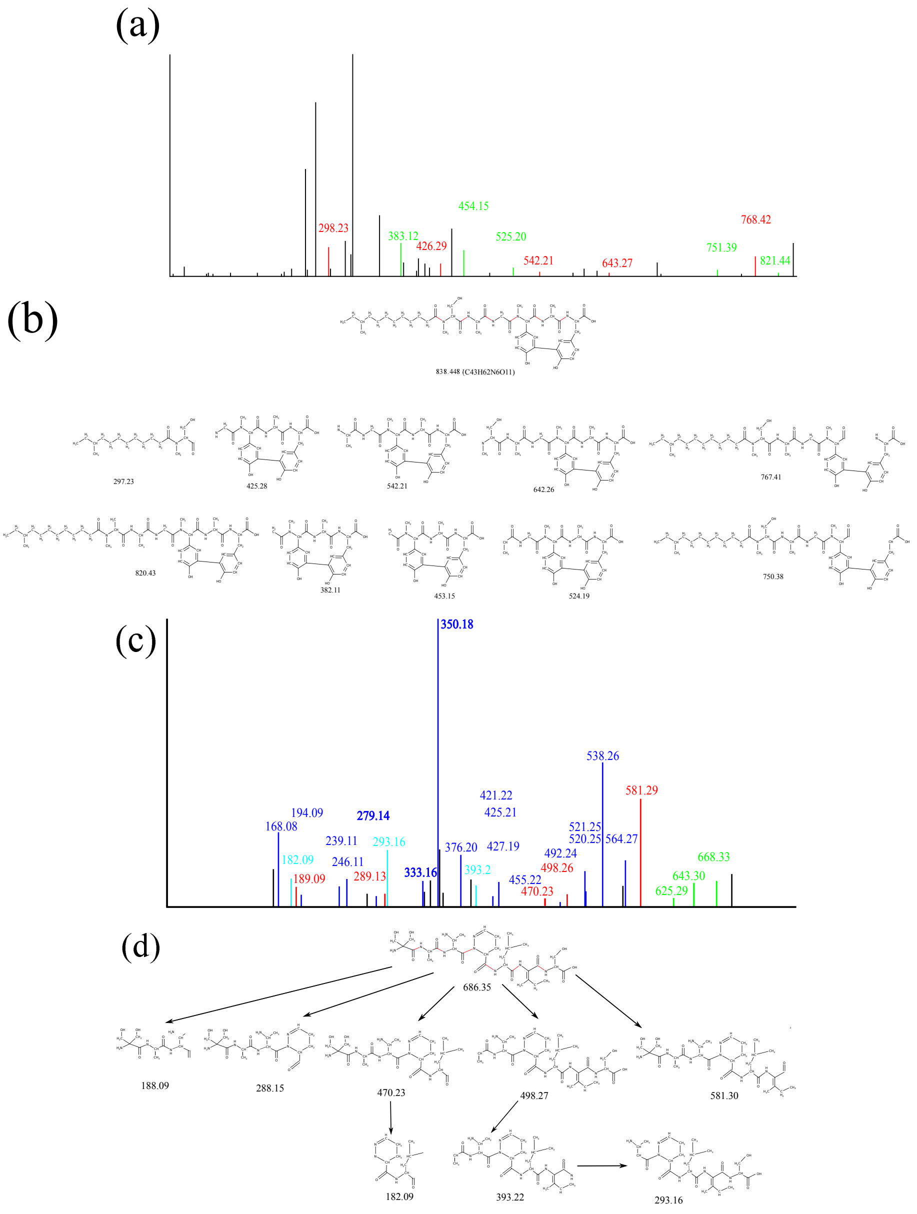

Supplementary Figure 3: Dereplicator+ annotation of arylomycin and antrimycin spectra. (a) Arylomycin mass spectra, (b) annotation of arylomycin mass spectra, (c) antrimycin mass spectra, and (d) annotation of antrimycin mass spectra. DEREPLICATOR annotations (amide bonds at depth 1) are shown in red, DEREPLICATOR-G annotations (general bonds at depth 1) are shown in green, DEREPLICATOR-PEP-FG annotations (amide bonds, depth 2 or more) are shown in light blue, DEREPLICATOR+ annotations (general bonds, depth 2 or more) are shown in dark blue. In case of Arylomycin we have 5 DEREPLICATOR annotations, and 6 more DEREPLICATOR-G annotations (total score 11). In case of antrimycin we have 5 DEREPLICATOR annotations, 3 DEREPLICATOR-PEP-FG annotations, 3 DEREPLICATOR-G annotations, and 16 additional DEREPLICATOR+ annotations (total score 27).

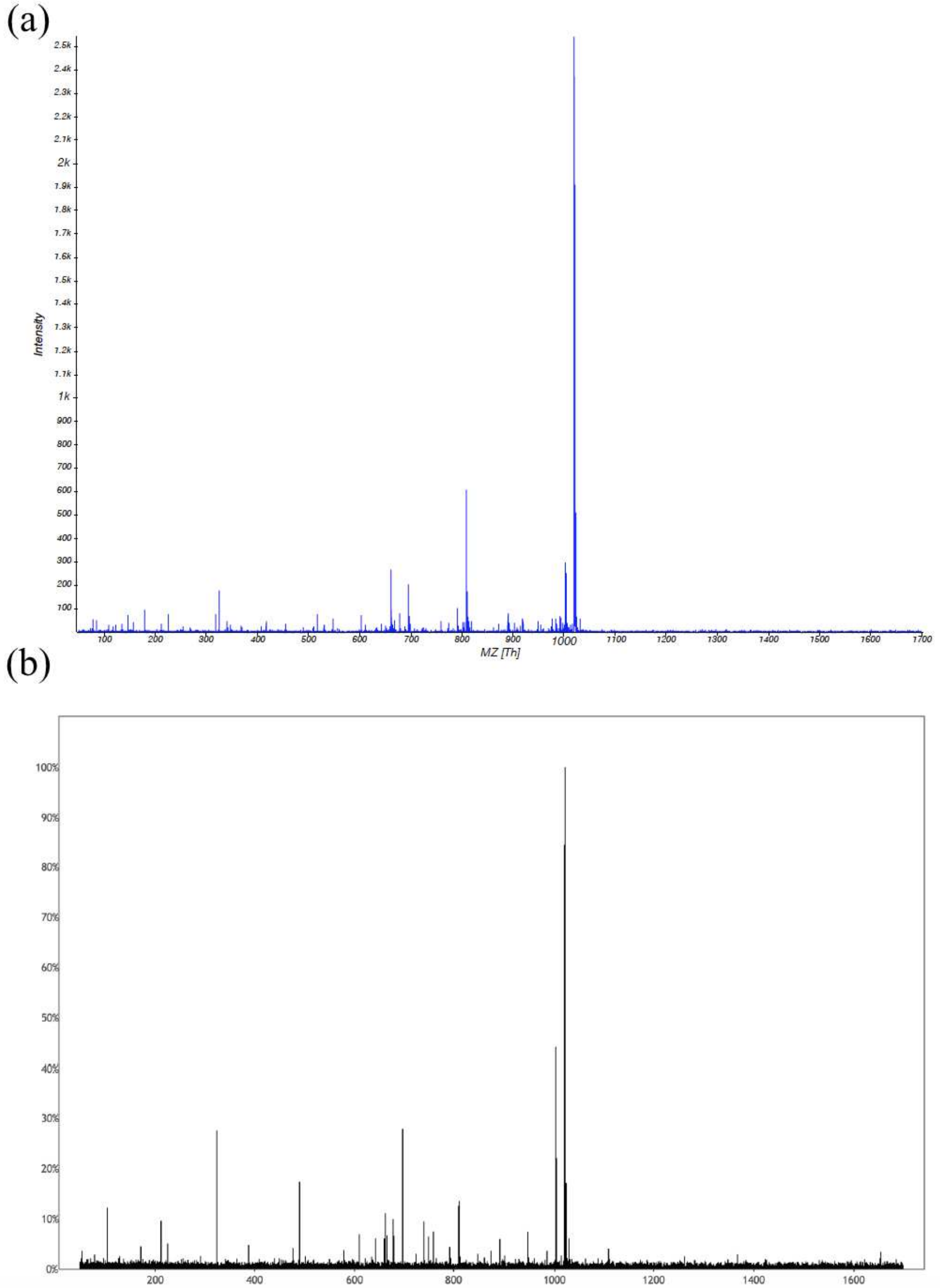

Supplementary Figure 4: Dereplicator+ identification of salinamide matches GNPS spectral library. (a) Spectra of salinamide identified by DEREPLICATOR+ in $\text{Spectra}_{\text{ActiSeq}}$. (b) Spectra of Salinamide from GNPS spectral library.

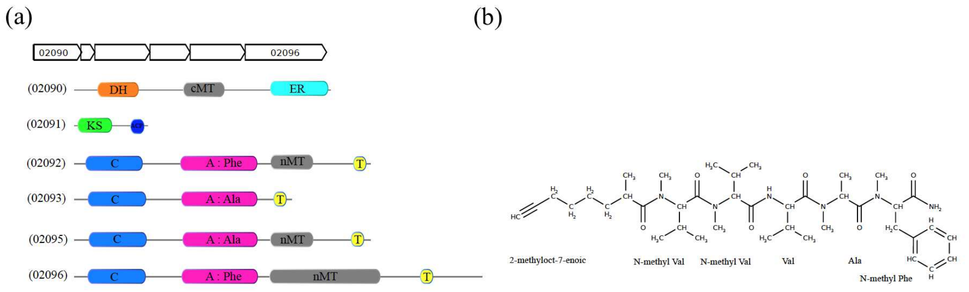

Supplementary Figure 5: Annotation of almiramide biosynthetic gene cluster. (a) Putative almiramide biosynthetic gene cluster starts with a PKS domain and consists adenylation domains encoding a (methylated) phenylalaline, an alaline, a (methylated) alanine, and a (methylated) phenylalaline. (b) The structure of almiramide B.

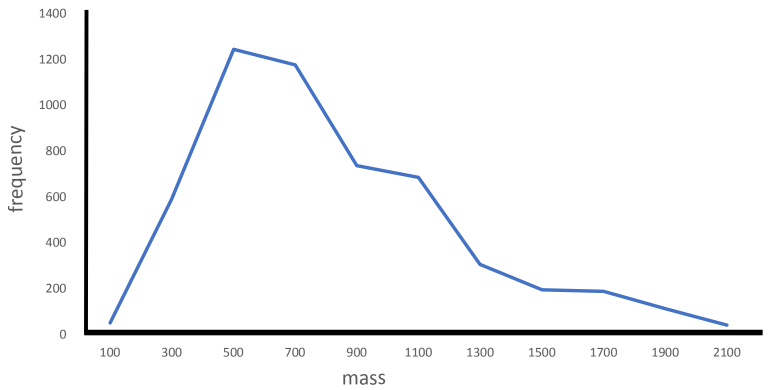

Supplementary Figure 6: Distribution of the masses for 5336 identifications from whole GNPS.

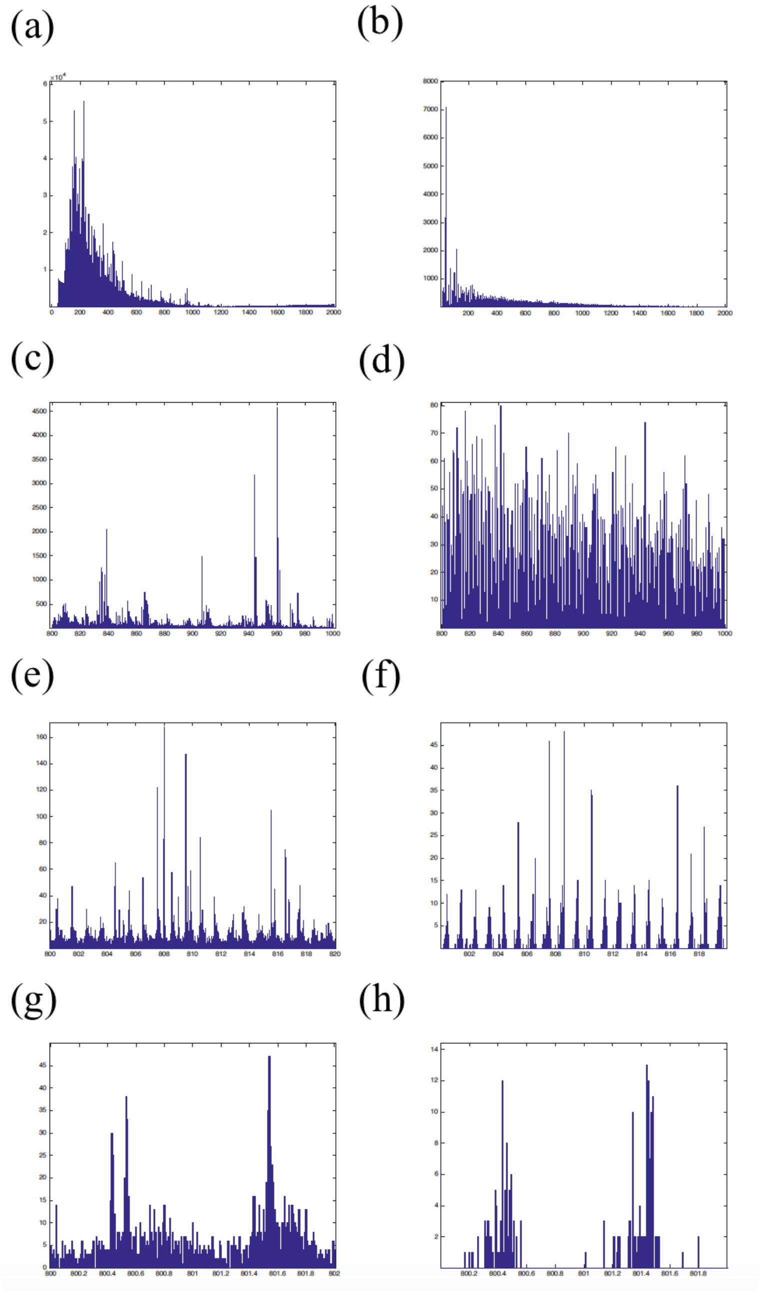

Supplementary Figure 7: The histograms of experimental and theoretical spectra of $\text{Spectra}_{\text{Lichen}}$ and AntiMarin. The histogram of (a) masses of all peaks from experimental spectra in $\text{Spectra}_{\text{lichen}}$ and (b) masses of all peaks in theoretical spectra from AntiMarin dataset in the 0-2000 Da interval. (c,d) similar distribution focused on 800-1000 Da interval. (e,f) similar distribution focused on 800-820 Da interval. (g,h) similar distribution focused on 800-802 Da interval.

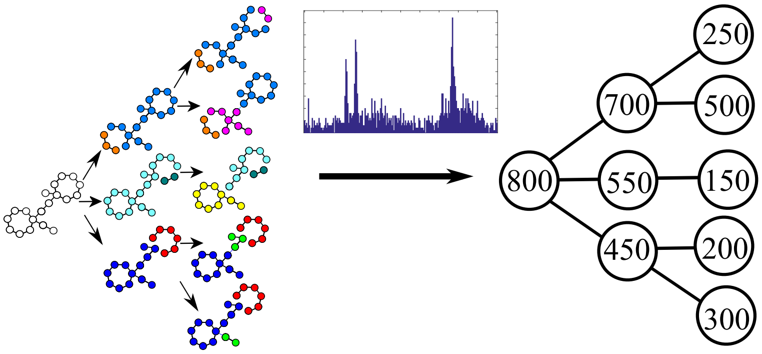

Supplementary Figure 8: Generating decoy fragmentation graph. For each target fragmentation graph, DEREPLICATOR+ generate a decoy fragmentation graph with the same structure and assigns masses to its nodes in a breadth-first manner as follows. The mass of the root is equal to the total mass of the metabolite, and for each node v, DEREPLISCATOR+ samples $mass(v)$ from the range $[0, mass(Parent(v))]$ of the distribution of all theoretical peaks, where $Parent(v)$ is the parent of node v.

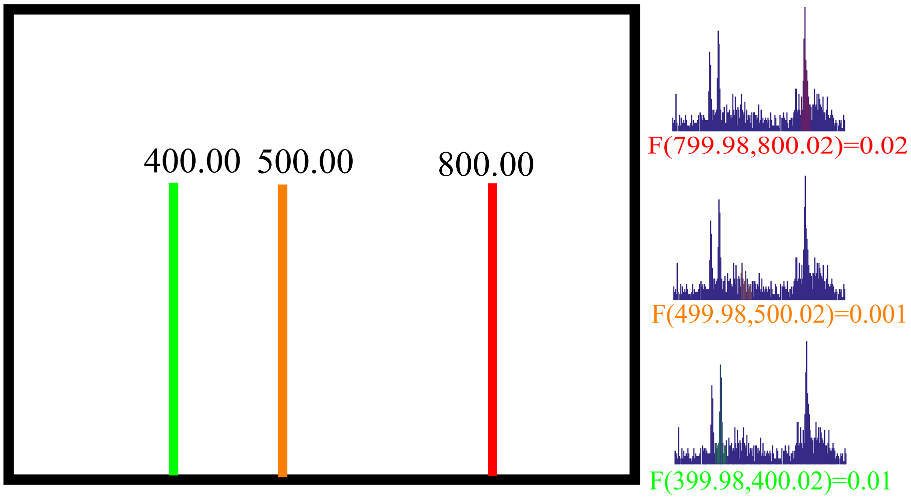

Supplementary Figure 9: Computing the statistical signicance of MSMs. Figure shows a depth-1 fragmentation graph with three peaks at masses 400.0, 500.0 and 800.0. For each peak with mass m, the probability $p(m)$ that a random experimental peak sampled from range $[0, M]$ of the experimental distribution annotates peak m within tolerance δ can be computed as $p(m) = F(m - \delta, m + \delta)$.

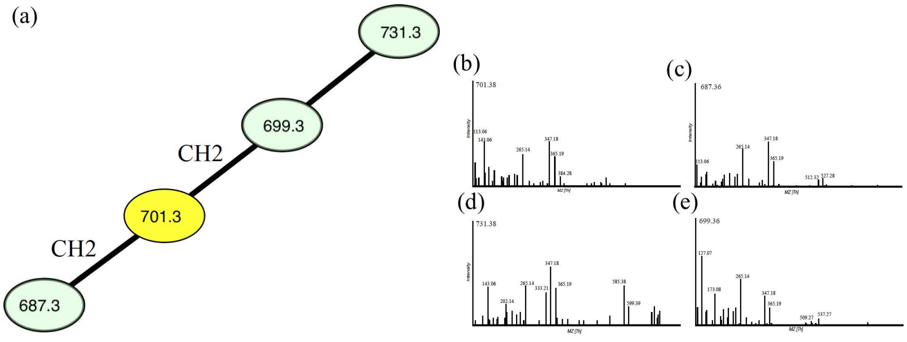

Supplementary Figure 10: The molecular networks of the chalcolmycin family. (a) Chalcomycin (shown in yellow) is identied by DEREPLICATOR+. Novel chalcomycin variants are shown in green. Mass spectra of (b) chalcomycin, (c) chalcomycin variant with mass 687, (d) chalcomycin variant with mass 731, (e) chalcomycin variant with mass 699. $CH2$ stands for methylation/demethylation mass difference.

SUPPLEMENTARY TABLES

Supplementary Table 1. Information about spectral datasets.

dataset	number of spectra	GNPS IDs
$\text{Spectra}_{\text{GNPS}}$	≈ 248,000,000	548 datasets
$\text{Spectra}_{\text{Fungi}}$	2475907	MSV000079098
$\text{Spectra}_{\text{Acti}}$	5598651	MSV000078787, MSV000078936, MSV000078937, MSV000078836, MSV000078839
$\text{Spectra}_{\text{Pseu}}$	3529556	MSV000078803, MSV000078817, MSV000078635, MSV000078606
$\text{Spectra}_{\text{Cyan}}$	11921457	MSV000078568
$\text{Spectra}_{\text{Acti-seq}}$	178635	MSV000078604, MSV000078839
$\text{Spectra}_{\text{Lichen}}$	926864	MSV000078584
$\text{Spectra}_{\text{library}}$	5473	-

Supplementary Table 2. Gene cluster of candidate Almiramide.

most similar gene	coverage	identity	length
lipid-A-disaccharide synthase	64%	77%	645
cobaltochelatase CobN subunit	100%	86%	1330
serine/threonine protein kinase	92%	73%	284
jamJ	95%	77%	1336
jamJ	98%	72%	383
tubC protein	99%	46%	1545
PuwG	94%	55%	1135
tubC protein	99%	45%	1546
CrpC	64%	57%	2303
2-hydroxy-6-oxohepta-2,4-dienoate hydrolase	100%	100%	293
methyltransferase	100%	100%	243
LmbE-like protein	96%	63%	279
glycosyltransferase	100%	99%	694

Supplementary Table 3. The most frequent atoms in all metabolites from the AntiMarin database. $freq_{marin}$ and $freq_{terres}$ stand for frequencies of different atoms within marine and terrestrial compounds.

atom	frequency	$freq_{marin}$	$fereq_{terres}$
H	2272193(51.7%)	217513(51.7%)	2054680(51.7%)
C	1597940(36.3%)	153866(36.6%)	1444074(36.3%)
O	400204(9.1%)	37804(8.8%)	362400(9.1%)
N	97945(2.2%)	8403(1.9%)	89542(2.2%)
S	8151(0.2%)	842(0.2%)	7309(0.2%)
Br	8002(0.2%)	1403(0.3%)	6599(0.2%)
Cl	6195(0.2%)	824(0.2%)	5371(0.2%)

Supplementary Table 4. The most frequent bonds in all metabolite structures from the AntiMarin database. $freq_{marin}$ and $freq_{terres}$ stand for frequencies of different bonds within marine and terrestial compounds.

bond	frequency	$freq_{marin}$	$freq_{terres}$
C-H	2050594(45.4%)	196820(45.6%)	1853774(45.4%)
C-C	1283757(28.4%)	121283(28.1%)	1162474(28.4%)
C-O	356218(7.8%)	34168(7.9%)	322050(7.8%)
C=C	222100(4.9%)	23698(5.4%)	198402(4.9%)
C-N	183013(4.0%)	16505(3.8%)	166508(4.0%)
C=O	140625(3.1%)	11868(2.7%)	128757(3.1%)
O-H	138568(3.0%)	14552(3.3%)	124016(3.0%)
N-H	82728(1.8%)	6130(1.4%)	76598(1.8%)
C=N	11747(0.2%)	1109(0.2%)	10638(0.2%)
C-S	9179(0.2%)	998(0.2%)	8181(0.2%)
C-Br	7998(0.1%)	1403(0.3%)	6595(0.1%)
C-Cl	6191(0.1%)	824(0.1%)	5367(0.1%)
S=O	5007(0.1%)	447(0.1%)	4560(0.1%)

SUPPLEMENTARY NOTES

Supplementary Note 1. Datasets.

MSV000078839 dataset. 36 strains of Streptomyces were grown on A1, MS and R5 agar, extracted sequentially with ethyl acetate, butanol and methanol, and analyzed on Agilent 6530 Accurate-Mass Q-TOF spectrometer coupled to a C18 RP Agilent 1260 LC system (ESI ionization, CID fragmentation).

MSV000078604 dataset. 16 strains of Streptomyces were grown on ISP2 agar plates, extracted by butanol, and analyzed on LTQ Orbitrap Velos coupled to a C18 RP Agilent 1200 LC system (ESI ionization, HCD fragmentation).

MSV000078568 dataset. A total of 317 cyanobacterial collections were extracted repetitively with CH2Cl2:MeOH 2:1, dried in vacuo, and fractionated into nine fractions (A-I) by silica gel vacuum liquid chromatography (VLC) using a stepwise gradient of hexanes/EtOAc and EtOAc/MeOH, and analyzed on a Maxis Impact mass spectrometer coupled to C18 RP-UHPLC (ESI ionization, CID fragmentation).

MSV000078584 dataset. Metabolites from 110 spots of lichen were extracted with 4:1 ethyl acetate-methanol and 0.1% trifluoroacetic acid (TFA), and analyzed on Maxis Q-TOF mass spectrometer (Bruker Daltonics) coupled to a C18 RP UltiMate 3000 UHPLC (ESI ionization, CID fragmentation).

Supplementary Note 2. Further analysis of $\text{Spectra}_{\text{ActiSeq}}$ dataset.

We divided $\text{Spectra}_{\text{ActiSeq}}$ into two parts, $\text{Spectra}_{\text{ActiSeq-CID}}$ consisting of 473135 spectra from MSV000078839 dataset and $\text{Spectra}_{\text{ActiSeq-HCD}}$ consisting of 178635 spectra from MSV000078604 dataset. DEREPLICATOR+ identified 2979 spectra (129 compounds) in $\text{Spectra}_{\text{ActiSeq-CID}}$ and 5215 spectra (404 compounds) in $\text{Spectra}_{\text{ActiSeq-HCD}}$. 45 compounds are found in both CID and HCD datasets. Supplementary Data 1 shows compounds discovered in CID and HCD spectra at 1% FDR.

To evaluate whether DEREPLICATOR+ incorrectly identifies common mass spectrometry contaminants as natural products, we performed an evaluation of the masses of 760 contaminants from Keller et al.

Among these 760 masses, 319 are present at 0.02Da threshold in $\text{Spectra}_{\text{ActiSeq}}$. However, only m/z 1020.50 (bovine trypsin) identified by DEREPLICATOR+ and reported as Salinamide A at 1% FDR (Supplementary Data 3). We manually confirmed that spectra at m/z 1020.50 is indeed Salinamide A and not a contaminant (Supplementary Figure 4).

DEREPLICATOR+ search of $\text{Spectra}_{\text{ActiSeq}}$ against AntiMarin, HMDB, LipidMaps, DNP, DrugBank, GNPS spectral library, KEGG, MiBIG, StreptomeDB, and UNPD identified 539 compounds at 1% FDR (Supplementary Data 4).

Supplementary Note 3. Further analysis of $\text{Spectra}_{\text{library}}$ dataset.

Compounds in $\text{Spectra}_{\text{Library}}$ have on average 8 isomers with identical chemical formula in DNP. Among 4360 compounds (80%) of $\text{Spectra}_{\text{Library}}$, which have at least one other isomer in the DNP database, DEREPLICATOR+ correctly identified 1746 (40%) of compounds (Supplementary Data 6).

To assess the capability of DEREPLICATOR+ in identifying adducts, we searched 1207 annotated spectra with sodium/potassium adducts from the GNPS spectral library using DEREPLICATOR+, and 280 (23%) of them were correctly identified at 1% FDR, while 23 (2%) were falsely identifies as a non-adduct compounds (Supplementary Data 8).

To assess the capability of DEREPLICATOR+ in identifying compounds in the negative ionization modes, we analyzed 341 additional spectra in the GNPS spectral library collected in the ESI (-) mode. DEREPLICATOR+ search correctly identified 88 (26%) of these compounds as top predictions (Supplementary Data 9).

Supplementary Note 4. Fragmentation model selection. To evaluate the rationality of the rules and thresholds in the fragmentation model, we compared 14 fragmentation models with various parameters used in conditions (i)-(iv). The comparison is based on the log-likelihood of these models in explaining the experimental spectrum (in comparison to the null model) over a set of 5473 Metabolite-Spectrum Matches (MSMs) from the $\text{Spectra}_{\text{library}}$ dataset. The models differ from each other in the chemical bonds they fragment, the maximum number of bridges and 2-cuts they allow, and whether or not they allow for the breakage of C-C 2-cuts and multiple C-C bridges. $\#Max_L$ stands for the maximum number of bridge fragmentations allowed. $\#Max_C$ stands for the maximum number of the 2-cut fragmentations allowed. $\#Max_{2L+C}$ is an upper-bound on twice the number of 2-cuts plus the number of bridges (i.e. total number of cuts in the fragmentation). $theo_{ef}$ stands for the size of the effective theoretical spectrum of a MSM, defined as peaks in the theoretical spectrum with annotated parents (the root is always annotated). e stands for the number of explained peaks in the experimental spectrum. For a specific model θ, p_θ stand for the probability of a peak in the effective theoretical spectrum being explained by the model. The null hypothesis assumes peaks are explained independent of the theoretical spectrum, with a constant probability p_{null}. LL stands for log-likelihood, defined as the log-ratio of the probability of the match under the model, to that of the match under the null hypothesis.

Given a set L MSMs $(M_1, S_1), \cdots (M_L, S_L)$, the log-likelihood score LL for a model θ is defined as :

$$LL_\theta = \sum_{t=1}^{L} LL_\theta(M_t, S_t) \tag{1}$$

where

$$LL_\theta(M, S) = log\frac{p_\theta(M, S)}{p_{null}(M, S)} = e * log\frac{p_\theta}{p_{null}} + (theo_{ef} - e) * log\frac{1 - p_\theta}{1 - p_{null}} \tag{2}$$

Parameters p_θ is approximated as the probability that a peak in the effective theoretical spectra is explained by a peak in the corresponding theoretical spectra, while p_{null} is approximated as the probability that a peak in the effective theoretical spectra is explained by a peak in a random spectra.

Supplementary Data 14 shows that addition of the OC and CC bonds increased the log-likelihood score from 7051 to 16054. From depth one to two, the log-likelihood score increased from 16054 to 27121. From depth two to depth three, the log-likelihood score increased from 27121 to 29888. At depth three, allowing for breakage of CC 2-cuts bonds or multiple CC bridges decreased the log-likelihood by 8% (from 29888 to 27565). Increasing the depth from 3 to 6 increased the log-likelihood from 29888 to 32394. Allowing for more than a single 2-cut breakage at depth 6 decreased the log-likelihood by a factor of 3% (from 32394 to 31404). Further increasing the depth from 6 to 9 hardly improved the log-likelihood (from 32394 to 32401). Our results show that, for condition (i), the optimal value of k is 6, and conditions (ii), (iii) and (iv) are crucial to maintain a fragmentation graph consistent with mass spectrometry fragmentation.
