# Supplementary material for: Dereplication of microbial metabolites through database search of mass spectra
Source: Nat Commun. 2018 Oct 2;9:4035. doi: 10.1038/s41467-018-06082-8 (PMC6168521; doi:10.1038/s41467-018-06082-8)
Supplement: Supplementary file 3 — Description of Additional Supplementary Files [file 41467_2018_6082_MOESM3_ESM.pdf]

Description of Additional Supplementary Files

Supplementary Data 1
Description: List of 488 top identifications of DEREPLICATOR+ from $Spectra_{ActiSeq}$.

Supplementary Data 2
Description: For a set of 207 DNP compounds from DNP annotated as ”Actinomyces/Streptomyces”, 56 are captured by DEREPLICATOR+.

Supplementary Data 3
Description: Among these 760 frequent mass spectrometry contaminations, the mass of 319 are present at 0.02Da threshold in $Spectra_{ActiSeq}$ database. However, only m/z 1020.50 (bovine trypsin) is among the identifications of DEREPLICATOR+, reported as Salinamide A at 1% FDR threshold. We manually confirmed that spectra at m/z 1020.50 is indeed Salinamide A and not a contaminant.

Supplementary Data 4
Description: DEREPLICATOR+ search of $Spectra_{ActiSeq}$ against AntiMarin, HMDB, LipidMaps, DNP, DrugBank, GNPS spectral library, KEGG, MiBIG, StreptomeDB, and UNPD, identified 539 compounds at 1% FDR.

Supplementary Data 5
Description: (a) At 1% FDR DEREPLICATOR+ recovered 13/20 identifications from NIST, 8/12 identifications from LipidBlast, and 11/16 identifications from MoNA (shown in yellow, green and red, respectively). At this FDR threshold DEREPLICATOR+ identified total of 315 compounds, 272 of them absent from LipidBlast, MoNA and NIST search results. (b) DEREPLICATOR missed 7, 4 and 5 compounds from LipidBlast, MoNA and NIST.

Supplementary Data 6
Description: List of DEREPLICATOR, DEREPLICATOR-g and DEREPLICATOR+ identifications in $Spectra_{library}$ at precursor mass tolerance of 5 Da.

Supplementary Data 7
Description: Performance of MS-Finder on *Spectra*$_{library}$. 3318 out of 5473 compounds were successfully analyzed by MS-Finder, running on a Dell Precision Tower 5810 with operating system Microsoft Windows 10 Enterprise. MS-FINDER failed to analyze the rest of compounds due to memory consumption. Among the successful runs, MS-FINDER correctly identified 677 (20%) of the correct compounds as top identification, 920 (27%) among top three, and 1274 (38.3%) among top ten identifications.

Supplementary Data 8
Description: Performance of DEREPLICATOR+ in identification of compounds with sodium/potassium adducts.

Supplementary Data 9
Description: Performance of DEREPLICATOR+ in identification of compounds with negative charge.

Supplementary Data 10
Description: List of 21 top identifications of DEREPLICATOR+ from *Spectra*$_{Lichen}$.

Supplementary Data 11
Description: List of 790 top identifications of DEREPLICATOR+ from *Spectra*$_{Cyano.}$

Supplementary Data 12
Description: List of 5336 top identifications of DEREPLICATOR+ from *Spectra*$_{GNPS}$.

Supplementary Data 13
Description: (a) Among GNPS datasets (317.2 million spectra) in Spectra$_{\mathrm{GNPS}}$ 72.7% (230.1 million) are from TOF instruments, while 18.1% (57.5) million are from Orbitrap instruments. Among identifications in *Spectra*$_{GNPS}$, 4.4 million (62.0%) are from TOF and 2.3 million (32.8%) are from Orbitrap instruments. (b) Number of sample, spectra, identified spectra, and instrument for each of the 555 GNPS datasets analyzed.

Supplementary Data 14

Description: Log-likelihood probability for 14 different fragmentation models. The models differ from each other in the chemical bonds they fragment, the maximum number of bridges and 2-cuts they allow, and whether or not they allow for the breakage of C-C 2-cuts are multiple C-C bridges. $\#Max_L$ stands for the maximum number of bridge fragmentations allowed. $\#Max_C$ stands for the maximum number of the 2-cut fragmentations allowed. $\#Max_{2L+C}$ is an upper-bound on twice the number of 2-cuts plus the number of bridges (i.e. total number of cuts in the fragmentation). $theo_{ef}$ stands for the size of the effective theoretical spectrum of a MSM, defined as peaks in the theoretical spectrum with annotated parents (the root is always annotated). *#explained* stands for the number of explained peaks in the experimental spectrum. For a specific model θ, p_θ stand for the probability of a peak in the effective theoretical spectrum being explained by the model. The null hypothesis assumes peaks are explained independent of the theoretical spectrum, with a constant probability p_{null}. *LL* stands for log-likelihood, defined as the log-ratio of the probability of the match under the model, to that of the match under the null hypothesis.
